# Impact of Coffee Intake on Sleep Quality Among Adults in the Jazan Region, Saudi Arabia: A Cross-Sectional Study

**DOI:** 10.7759/cureus.93386

**Published:** 2025-09-28

**Authors:** Mohammad A Jareebi, Waleed A Alabdali, Fahad A Alwan, Albaraa I Ageeli, Feras A Akoor, Feras Y Ageeli, Hussam A Alhazmi, Mohammed A Alfifi, Dhiyaa A Otayf, Majed A Ryani, Ahmed A Bahri, Mohammed A Muaddi, Abdullah A Alharbi, Yahya H Khormi, Ahmad Y Alqassim

**Affiliations:** 1 Family and Community Medicine Department, Jazan University, Jazan, SAU; 2 College of Medicine, Jazan University, Jazan, SAU; 3 Surgery Department, Jazan University, Jazan, SAU

**Keywords:** caffeine, coffee, jazan region, lifestyle, saudi arabia, sleep quality

## Abstract

Background: Coffee consumption is a widespread habit globally, and especially among Saudis. However, its association with sleep quality remains unclear. This study aimed to evaluate the effect of coffee consumption on sleep quality among adults in Jazan, Saudi Arabia.

Methods: This cross-sectional study utilized an electronic survey targeting residents of the Jazan region aged 18 years and older. Melatonin users, those on caffeine-restricted diets, and non-consenting participants were excluded. The survey included demographic data, coffee intake patterns, and the Sleep Quality Scale (SQS).

Results: Among the 824 participants, 695 (84%) reported consuming coffee, with a mean daily intake of 2.24 ± 1.61 cups. Good sleep quality was reported by 92% of participants (mean SQS score: 41 ± 14). No significant association was found between coffee intake and sleep quality (daily drinker: β = 1.66, p = 0.415; regular drinker: β = 2.72, p = 0.151). However, males had better sleep quality (β = -4.17, p < 0.001). In contrast, military occupation (β = 6.95, p = 0.019), longer electronic device use (β = 0.39 per hour, p = 0.001), ex-smoking (β = 4.58, p = 0.010), and khat use (β = 4.84, p = 0.034) were associated with poorer sleep quality.

Conclusions: Despite high coffee consumption in Jazan, no significant relationship was observed between coffee intake and sleep quality. However, gender, occupation, electronic device usage, smoking, and khat chewing were significant factors. Future studies should incorporate objective measures and target individuals at risk.

## Introduction

Coffee, a widely consumed beverage with a longstanding tradition and cultural value, owes its origin to Ethiopia by a herder named Kaldi, who discovered that his goats displayed greater strength after consuming plant berries containing caffeine, which were used to make coffee beans [1]. As a demonstration of this, caffeine is a xanthine alkaloid molecule that is a potent central nervous system (CNS) stimulant found in many beverages and medications. The primary physiological impact of coffee is CNS stimulation, which frequently raises arousal and alertness after drinking it [2]. Regarding coffee consumption, it has become a daily necessity for individuals worldwide. Approximately 530 million liters, or 2.25 billion cups, of coffee are used daily worldwide, an incredible amount of coffee [3]. Saudi Arabia is one of the world's top 10 countries for coffee consumption. In Saudi Arabia, coffee is consumed by nine out of ten people. Even though coffee is an essential source of energy and alertness for millions of people worldwide, its use needs to be balanced with the possibility that it may have a negative impact on sleep quality and overall fitness [4].

Since individuals spend about a third of their lives sleeping, it is important for their overall well-being. Just as food and water are necessary for life, so is the amount and quality of sleep. It plays a significant role in the growth and operation of the neural connections in your brain that support memory and learning processes [5]. Additionally, the sleep and wake cycle generated by inherent brain networks is controlled by the biological clock. Sleep onset and maintaining it require suppressing ascending arousal mechanisms that promote awakening. The waking episode ends when extracellular adenosine levels increase, indicating the initiation of sleep. Adenosine serves as a sleep control by stimulating the inhibitory neurons in the brain's ventrolateral pre-optic area [6]. It is estimated that 30% of adults globally suffer from sleep deprivation, sleeplessness, and other sleep-related issues [7]. In addition, insomnia and other sleep disorders are thought to affect 37.6% of Saudi Arabia's overall population [8]. Ultimately, attaining sufficient sleep is vital for mental stability and avoiding sleep conditions that affect a multitude of people globally.

Latest research provides insight into caffeine's possible consequences on acquiring proper sleep and emphasizes the puzzling correlation between coffee intake and sleep quality. For instance, a systematic review and meta-analysis conducted in 2023 by Gardiner et al. aimed to consider caffeine's impact on sleep across 24 studies. The study highlighted that the mean difference in the entire sleep interval for each group decreased by 2.8 minutes (95% confidence interval (CI) = 0.4 to 5.2 min) for every extra hour of coffee consumed before bed [5]. Likewise, Weibel et al. conducted a randomized controlled experiment in 2021. This sought to determine how regular morning and afternoon coffee intake affected the sleep pattern and sleep intensity at night after extended caffeine uptake during the day. According to the study findings, among healthy sleepers who frequently consume coffee, daily consuming coffee in the morning and afternoon did not considerably impact overnight sleep pattern or subjective sleep quality (P, all > 0.05) [9]. In Saudi Arabia, Khan and Alqurashi carried out a cross-sectional study in Makkah in 2024. Their participants reported high Pittsburgh Sleep Quality Index (PSQI) scores (median PSQI of 6 for > 400 mg vs. 5 for < 400 mg, p < 0.001), which is linked to poor sleep quality. Based on the study's findings, excessive coffee consumption and poor sleep quality are significantly associated (p < 0.001) [10,11].

Although the consumption of coffee and sleep disturbances are common worldwide, only a few studies have been done on this subject in Saudi Arabia [4,10,12]. Any research results are difficult to generalize due to their small sample sizes, narrow study areas, and failure to consider relevant sociodemographic variables. Furthermore, no study had specifically examined the effect of coffee consumption on sleep quality in the Jazan region. This represented an important gap, as the region is known for its unique cultural traditions and social practices surrounding coffee drinking. Coffee has deep cultural significance in Jazan, where its preparation and consumption are embedded in daily life and social gatherings, making it an integral part of the community’s lifestyle.

Given these factors, the intent of this research was to evaluate the effect of coffee intake on the quality of sleep among individuals aged 18 years and above in the Jazan region of Saudi Arabia. Specifically, the study examined the association between coffee consumption (frequency, daily intake, and timing) and Sleep Quality Scale (SQS) scores. By focusing on a population with a high prevalence of coffee consumption due to cultural traditions, this study provided context-specific insights into how coffee consumption affected sleep quality across different sociodemographic groups in Jazan.

## Materials and methods

Study design and participants

This cross-sectional, questionnaire-based study was carried out in the Jazan region between September 2024 and April 2025. The analysis included all residents of the Jazan region aged 18 and above. Individuals who use melatonin, those restricted from consuming caffeine (e.g., individuals with heart disease), or those who did not give permission were not recruited for the study. A non-random convenience sampling technique was used, and the sample size was evaluated using the formula given below:

$N=(Z^2\bullet P(1-P))/e^2$

where N is the required sample size, Z represents the desired confidence level (95%, equivalent to 1.96), P is the estimated proportion (50% or 0.5, using a conservative approach), and e is the margin of error (5%).

According to the latest census, the target population comprised 919,267 adults in the Jazan region aged 18 and above [13]. Based on these parameters, the minimum required sample size was 384 participants. However, 824 participants were recruited, aiming to enhance the accuracy and precision of the findings.

Data collection tool

A self-administered electronic questionnaire was employed based on a comprehensive literature review and a discussion with medical experts. The questionnaire comprised three sections: sociodemographic characteristics, coffee consumption, and the SQS [14].

The first section of the questionnaire obtained sociodemographic and health-related data, including age, sex, nationality, residence, education level, employment status, marital status, monthly household income, and family size. Health and lifestyle variables included weight, height, and body mass index (BMI, kg/m^2^; reported as mean ± standard deviation), smoking status (current, former, never), age at smoking initiation, smoking intensity (cigarettes/day), and use of shisha, vape, nicotine pouches, and khat (categorized as current, former, or never user). Additional variables included physical activity (none, moderate/vigorous ≥30 minutes on ≥5 days/week, or <30 minutes/<5 days/week), dietary habits (healthy/unhealthy), sleep aid use, chronic disease history, and daily electronic device exposure (hours/day).

The second section assessed coffee consumption using standardized measures. A "cup" was operationally defined as approximately 150 mL, regardless of coffee type (Arabic, espresso, instant, or brewed). Participants reported the number of cups consumed per day, type of coffee, preferred bean, brewing method, and decaffeinated status. Timing of intake was assessed by asking whether coffee was consumed during morning hours, evening hours, or both. Participants were also asked about the physiological effects of coffee (palpitations, insomnia, and withdrawal symptoms such as headaches, fatigue, irritability, and nausea). Additional items captured the intake of other caffeine sources, including tea, energy drinks, carbonated beverages, and chocolate.

The third section employed the SQS, a psychometrically validated instrument with high internal consistency (Cronbach’s α = 0.92) and acceptable test-retest reliability (r = 0.81). For the present investigation, the SQS was forward-translated into Arabic and subsequently reviewed by bilingual experts in sleep medicine and public health to ensure semantic, cultural, and conceptual equivalence prior to administration. The instrument comprises 28 items encompassing six domains: daytime symptoms, restoration after sleep, problems with sleep initiation and maintenance, difficulty waking, and sleep satisfaction. Items were rated on a 4-point Likert-type scale (0 = few, 1 = sometimes, 2 = often, 3 = almost always). Aggregate scores range from 0 to 84, with higher values reflecting poorer sleep quality. For categorical analyses, a cut-off score of ≥41 was applied to define poor sleep quality, consistent with prior validation studies [14]. Missing data were minimal (<5%) and addressed through pairwise deletion. The SQS is free to use for academic and research purposes [14].

The questionnaire was piloted with 60 people, reviewed, and minor language adjustments were made before being executed. The survey link was disseminated via widely used social media platforms in the Jazan region, including Twitter (X), WhatsApp, Telegram, Snapchat, and Facebook. Recruitment relied on voluntary participation through convenience sampling; therefore, no formal sampling frame was available to estimate a response rate, which may limit representativeness. Nevertheless, distribution across multiple platforms was intended to enhance diversity and mitigate clustering of responses within specific networks. The purpose and objectives of the research were explained to participants, and their permission was sought before inclusion in the study. Confidentiality was ensured during the analysis.

Data analysis

Descriptive statistics were reported as means with standard deviations for continuous variables and as frequencies with percentages for categorical variables. Sleep quality (SQS total score) was analyzed as a continuous outcome using multiple linear regression, with results expressed as β coefficients, 95% CIs, and p-values. Covariates included age, sex, BMI, employment status, marital status, income, smoking status, shisha use, vape use, nicotine pouch use, khat use, physical activity, diet, daily device exposure, and chronic disease history. Model assumptions (linearity, normality, and homoscedasticity) were assessed, and multicollinearity was examined using variance inflation factors. Missing data (<5%) were handled by pairwise deletion. All analyses were conducted using R software, version 4.2.3 (R Foundation for Statistical Computing, Vienna, Austria), with statistical significance set at p < 0.05.

Bias

To address selection bias due to the convenience sample resulting from the questionnaire, we distributed it across multiple social media platforms to diversify participation. Additionally, the relatively large sample size helped to reduce the bias. As for measurement bias, we conducted a pilot test for the questionnaire and used a validated, reliable, and comprehensive SQS [15].

Ethical considerations

Ethical approval was obtained from the Local Committee for Research Ethics at Jazan University (reference number REC-46/06/1285, dated 24 December 2024). All participants provided informed consent electronically after receiving a description of the study objectives, potential risks and benefits, and their rights to voluntary withdrawal, confidentiality, and privacy. Data collection was conducted between September 2024 and April 2025. Only the research team had access to de-identified data, and no personally identifiable information was collected. This study adhered to the principles of the Declaration of Helsinki and was reported in accordance with the Strengthening the Reporting of Observational Studies in Epidemiology (STROBE) guidelines [16]. Anonymized data and R code used for analysis are available from the corresponding author upon reasonable request.

## Results

Sociodemographic characteristics

Table 1 presents the sociodemographic characteristics of the sample, consisting of 824 participants. The mean age of participants was 33 years (SD = 13), and the mean family size was 6.7 family members per household (SD = 5.8). Regarding sex distribution, 344 (42%) were female and 480 (58%) were male. A total of 812 participants (99%) were Saudi nationals, while 12 (1%) were non-Saudis. In terms of residence, 463 (56%) lived in rural regions, whereas 361 (44%) resided in urban areas. For education level, 140 (17%) participants had a high school degree or lower, 653 (79%) held a bachelor’s or diploma degree, and 31 (4%) had a master’s or PhD degree. Regarding employment status, 402 (49%) were employed, 311 (38%) were students, and 111 (13%) were unemployed. Socially, 410 (49.76%) participants were non-married or engaged, 392 (47.57%) were married, and 22 (2.67%) were divorced or widowed. In terms of monthly income, 171 (21%) participants reported earning less than 5000 SAR, 140 (17%) earned between 5000 and 9999 SAR, 214 (26%) earned between 10,000 and 14,999 SAR, and 299 (36%) earned 15,000 SAR or more.

**Table 1:** Sociodemographic characteristics of study participants (n = 824) SD: standard deviation; n: sample size

Characteristics	Mean ± SD/ Frequency (%)
Age	33 ± 13 years
Family members	6.7 ± 5.8 kids/household
Sex	
Female	344 (42)
Male	480 (58)
Nationality	
Saudi	812 (99)
Non-Saudi	12 (1)
Residence	
Rural	463 (56)
Urban	361 (44)
Education	
High school degree or lower	140 (17)
Bachelor’s or diploma degree	653 (79)
Master’s or PhD	31 (4)
Employment	
Employed	402 (49)
Student	311 (38)
Unemployed	111 (13)
Marital status	
Non-married/engaged	410 (49.76)
Married	392 (47.57)
Divorced/widowed	22 (2.67)
Income	
Less than 5000	171 (21)
5000-9999	140 (17)
10000-14999	214 (26)
≥15,000	299 (36)

Lifestyle, smoking, and health characteristics

Table 2 shows lifestyle, smoking, and health metrics among 824 participants. The mean weight was 69.6 kg (SD = 18), height averaged 163.6 cm (SD = 9.9), and BMI was 26 (SD = 5.8). Participants reported a mean daily device usage of 7.7 hours (SD = 4.3). The average age for smoking initiation was 21 years (SD = 6.3), and smoking intensity averaged 13 cigarettes per day (SD = 11). Regarding physical activity, 317 (38%) reported no physical activity during the week, 329 (40%) were involved in moderate or vigorous physical activity for at least 30 minutes, five times a week, and 178 (22%) undertook such activity for less than 30 minutes, five times a week. Concerning dietary habits, 190 (23%) reported following a healthy diet, while 634 (77%) did not. For smoking habits, 70 (8.5%) participants were former cigarette smokers, 37 (4.5%) were current smokers, and 717 (87%) had never smoked cigarettes. Regarding shisha, 89 (10.8%) were former users, 62 (7.5%) were current users, and 673 (81.7%) had never smoked shisha. In terms of vaping, 65 (7.89%) were former users, 21 (2.55%) were current users, and 738 (89.56%) had never used vape products. For nicotine pouches (e.g., DZRT), 44 (5%) were former users, 40 (5%) were current users, and 740 (90%) had never used them. Concerning khat usage, 45 (5.5%) were former users, 45 (5.5%) were current users, and 734 (89%) had never used khat.

**Table 2:** Lifestyle, smoking, and health characteristics (n = 824) SD: standard deviation; n: sample size; BMI: body mass index; n: sample size; CPD: cigarettes per day

Characteristics	Mean ± SD/Frequency (%)
Weight	69.6 ± 18 kg
Height	163.6 ± 9.9 cm
BMI	26 ± 5.8
Daily device usage	7.7 ± 4.3 hours
Smoking initiation	21 ± 6.3 years
Smoking intensity	13 ± 11 CPD
Physical activity	
Yes	422 (51)
No	402 (49)
Physical activity type	
You do not engage in any physical activity during the week.	317 (38)
You engage in moderate or vigorous physical activity for at least 30 minutes, five times a week.	329 (40)
You engage in moderate or vigorous physical activity for less than 30 minutes, five times a week.	178 (22)
Healthy diet	
Yes	190 (23)
No	634 (77)
Smoking status	
Ex-smoker (former user)	70 (8.5)
Yes	37 (4.5)
No	717 (87)
Shisha status	
Ex-smoker (former user)	89 (10.8)
Yes	62 (7.5)
No	673 (81.7)
Vape status	
Former user	65 (7.89)
Yes	21 (2.55)
No	738 (89.56)
Nicotine pouches (e.g., DZRT) status	
Former user	44 (5)
Yes	40 (5)
No	740 (90)
Khat status	
Former user	45 (5.5)
Yes	45 (5.5)
No	734 (89)

Medical history and comorbidities

Table 3 presents the medical history and comorbidities of 824 participants. The data show that 71 (9%) participants had diabetes mellitus (DM), 75 (9%) had hypertension (HTN), and 84 (10%) had hyperlipidemia. Asthma was reported by 56 (7%) participants, while 19 (2%) had sickle cell disease (SCD) and 13 (2%) had thalassemia. Regarding thyroid conditions, 15 (2%) participants had hyperthyroidism, and 29 (4%) had hypothyroidism. Rheumatoid arthritis (RA) was reported by 27 (3%) participants, while 28 (3%) also reported anxiety. Depression was present in 34 (4%) participants, and 31 (4%) reported experiencing chronic pain. Finally, 284 (34%) participants had a vitamin D deficiency, while 540 (66%) did not have this condition.

**Table 3:** Distribution of Self-Reported Chronic Health Conditions (n = 824) SD: standard deviation; n: sample size; DM: diabetes mellitus; HTN: hypertension; SCD: sickle cell disease; RA: rheumatoid arthritis

Characteristics	Frequency (%)
DM	
No	753 (91)
Yes	71 (9)
HTN	
No	749 (91)
Yes	75 (9)
Hyperlipidemia	
No	740 (90)
Yes	84 (10)
Asthma	
No	768 (93)
Yes	56 (7)
SCD	
No	805 (98)
Yes	19 (2)
Thalassemia	
No	811 (98)
Yes	13 (2)
Hyperthyroidism	
No	809 (98)
Yes	15 (2)
Hypothyroidism	
No	795 (96)
Yes	29 (4)
RA	
No	797(97)
Yes	27 (3)
Anxiety	
No	796 (97)
Yes	28 (3)
Depression	
No	790 (96)
Yes	34 (4)
Chronic pain	
No	793 (96)
Yes	31 (4)
Vitamin D deficiency	
No	540 (66)
Yes	284 (34)

Coffee and caffeinated drinks habits

**Figure 1:**
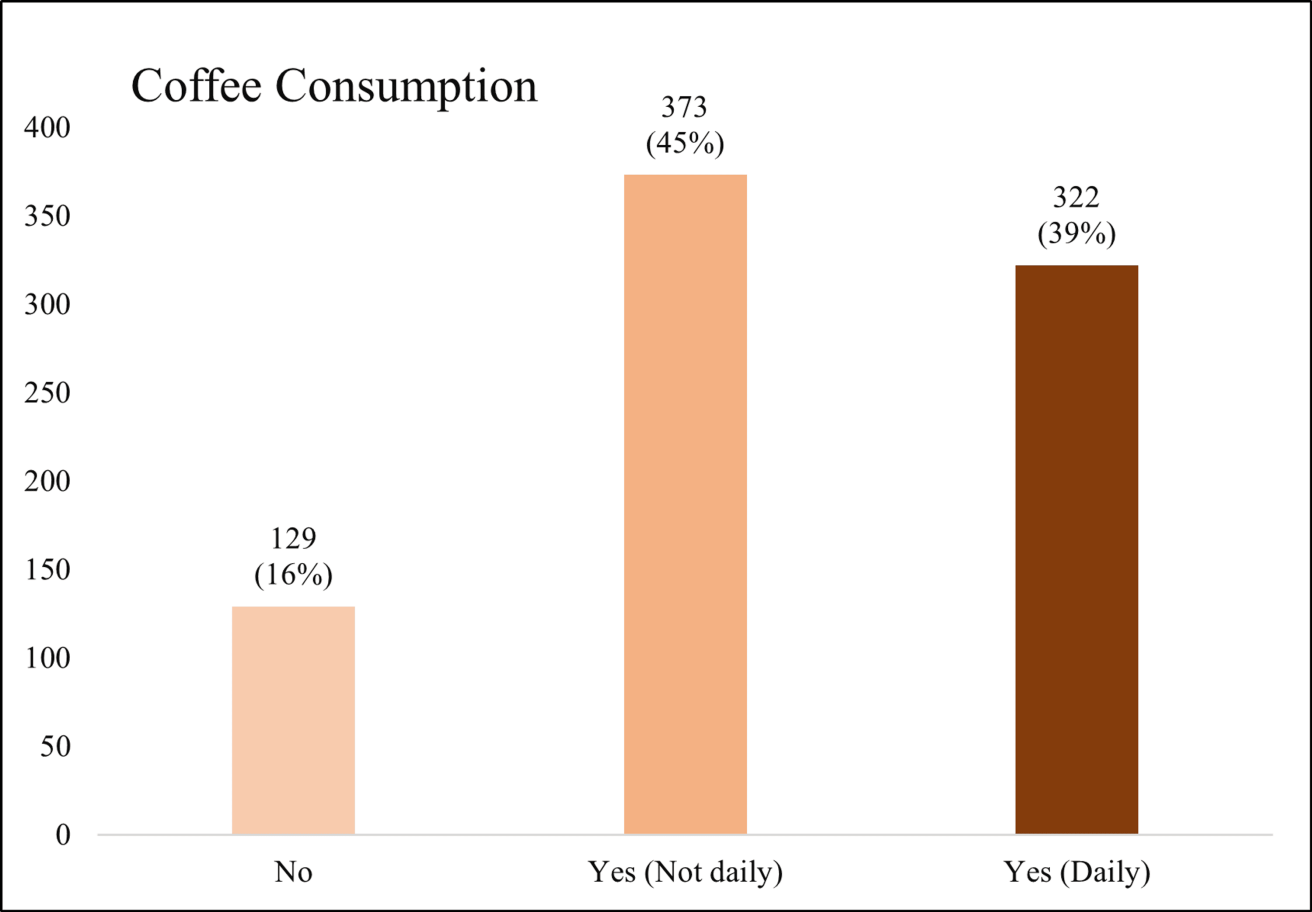
Coffee Consumption Frequency Among Participants The figure illustrates the distribution of coffee consumption among participants (n = 824). A total of 129 (16%) participants reported not consuming coffee, while 373 (45%) consumed coffee regularly but not on a daily basis. The remaining 322 (39%) of participants consumed coffee daily.

Table 4 presents coffee and caffeinated drink habits among 824 participants, reporting that the mean daily coffee consumption was 2.24 ± 1.61 cups. A total of 322 (39%) participants drank coffee daily, 373 (45%) drank it regularly but not daily, and 129 (16%) abstained entirely. Coffee consumption prevalence is nicely displayed in Figure 1. Preferences included black coffee for 306 (37%) participants, Saudi coffee for 276 (33%), coffee with milk for 92 (11%), and other types for 21 (3%), while 129 (16%) reported not drinking coffee. Regarding temperature preferences, 433 (52.55%) participants preferred hot coffee and 262 (31.90%) preferred cold coffee, while 129 (15.65%) did not drink coffee. Coffee consumption timing varied: 277 (33.62%) drank at no specific time, 139 (16.87%) in the morning, 133 (16.14%) in the evening, and 146 (17.72%) in both morning and evening, while 129 (15.65%) did not drink coffee. Among coffee drinkers (n = 695), caffeine-induced symptoms included restlessness in 194 (24%) and palpitations in 115 (14%), while caffeine deprivation symptoms included headaches in 182 (22%), fatigue in 155 (19%), irritability/anger in 98 (12%), and nausea in 66 (8%). Regarding other caffeinated beverages, soft drinks were consumed “always” by 214 (26%), “sometimes” by 430 (52%), and “never” by 180 (22%). Energy drinks were consumed “always” by 55 (7%), “sometimes” by 248 (30%), and “never” by 521 (63%). Tea consumption was reported as “always” by 348 (42%), “sometimes” by 410 (50%), and “never” by 66 (8%).

**Table 4:** Coffee and Caffeinated Drink Habits (n = 824) SD: standard deviation; n: sample size; * among coffee drinkers (n = 695)

Characteristics	Mean ± SD/Frequency (%)
Daily coffee consumption	2.24 ± 1.61 cups/day
Coffee	
Yes, on daily basis	322 (39)
Yes, regularly but not daily	373 (45)
Not a coffee drinker	129 (16)
Preferred coffee type	
Black coffee	306 (37)
Coffee with milk	92 (11)
Saudi coffee	276 (33)
Other types	21 (3)
Not a coffee drinker	129 (16)
Cold or hot coffee (often)	
Cold coffee	262 (31.90)
Hot coffee	433 (52.55)
Not a coffee drinker	129 (15.65)
Time of drinking coffee	
No specific time	277 (33.62)
In the evening	133 (16.14)
In the morning	139 (16.87)
In the morning and evening	146 (17.72)
Not a coffee drinker	129 (15.65)
Caffeine-induced symptoms*	
Palpitations	115 (14)
Restless	194 (24)
Caffeine deprivation symptoms*	
Headache	182 (22)
Fatigue	155 (19)
Irritability/anger	98 (12)
Nausea	66 (8)
Soft drink	
Always	214 (26)
Sometimes	430 (52)
No	180 (22)
Energy drinks	
Always	55 (7)
Sometimes	248 (30)
No	521 (63)
Tea	
Always	348 (42)
Sometimes	410 (50)
No	66 (8)

Sleep patterns and quality characteristics

Table 5 gives an account of sleep patterns and quality characteristics of the sample, which includes 824 participants. The mean sleep quality score was 41 (SD = 14), and the mean sleep duration was 6.9 hours (SD = 2). In terms of sleep quality, 759 (92%) participants were categorized as having good sleep quality, while 65 (8%) were categorized as having poor sleep quality.

**Table 5:** Sleep Patterns and Quality Characteristics (n = 824) SD: standard deviation; n: sample size

Characteristics	Mean ± SD/Frequency (%)
Sleep Quality Score	41 ± 14
Sleep duration	6.9 ± 2 hours/day
Sleep quality	
Good	759 (92)
Poor	65 (8)

Sleep score distribution

**Figure 2:**
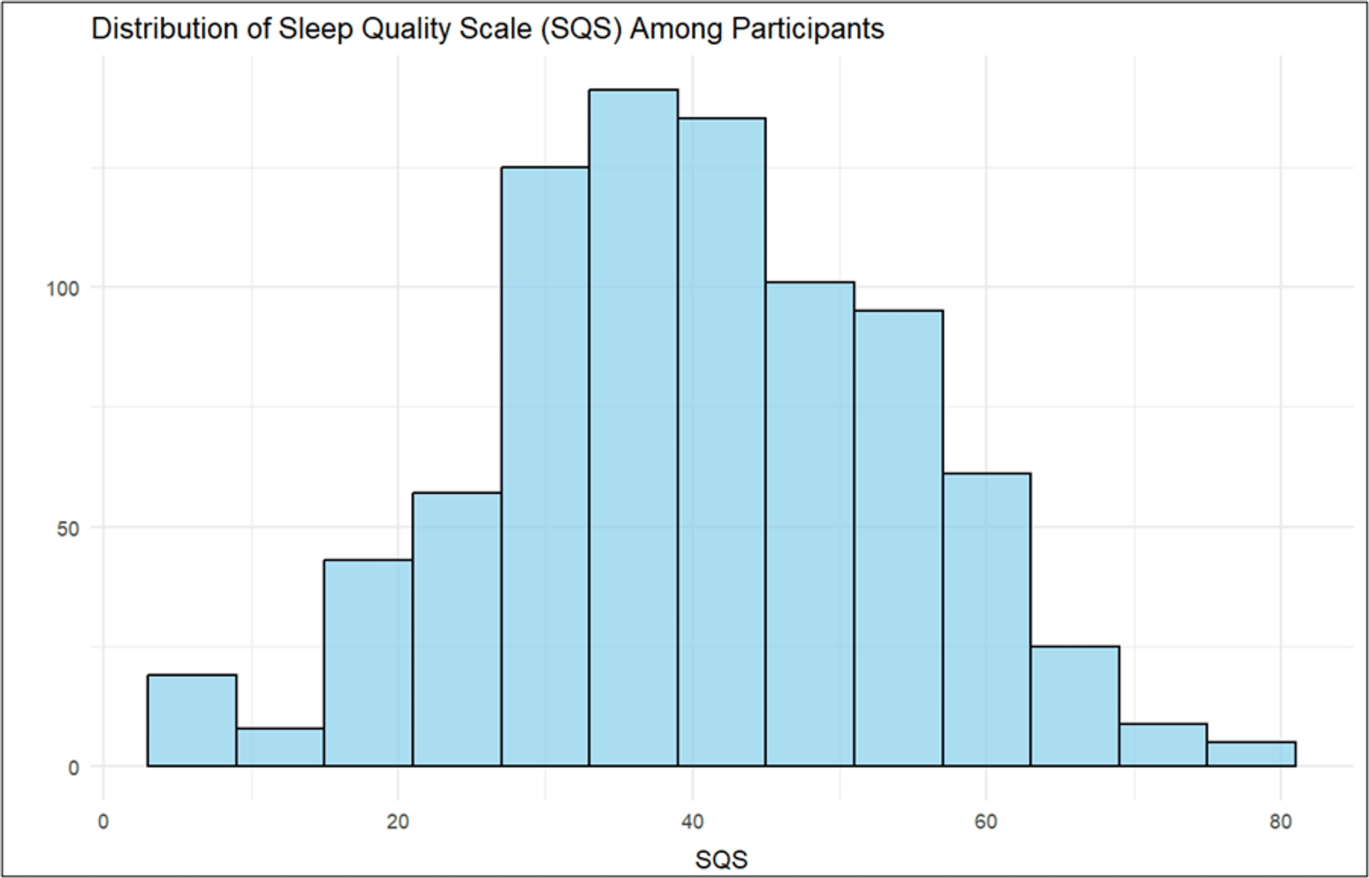
Sleep Quality Scale (SQS) Distribution

Figure 2 presents the distribution of SQS scores among participants. The mean score is 40.79 with an SD of 13.84, indicating moderate variability in the SQS (higher score indicates poorer sleep quality). The distribution appears to be approximately normal.

Impact of coffee intake and other study variables on sleep quality

Table 6 details different predictors of SQS among participants. Coffee consumption was associated with sleep quality, but these associations were not statistically significant. Neither daily coffee intake (β = 1.66, 95% CI: -2.34 to 5.67, p = 0.415) nor regular but non-daily consumption (β = 2.72, 95% CI: -0.99 to 6.43, p = 0.151). Similarly, coffee drinking time and daily consumption did not exhibit significant associations. Males reported better sleep quality compared to females (β = -4.17, 95% CI: -6.46 to -1.88, p < 0.001). Participants working in the military sector had poorer sleep quality than those in other sectors (β = 6.95, 95% CI: 1.14 to 12.76, p = 0.019). Reduced sleep quality was related to longer hours of device use (β = 0.39, 95% CI: 0.15 to 0.62, p = 0.001). Compared to non-smokers, ex-smokers reported lower-quality sleep (β = 4.58, 95% CI: 1.12 to 8.05, p = 0.010). Additionally, individuals who used khat had poorer sleep quality compared to non-users (β = 4.84, 95% CI: 0.37 to 9.30, p = 0.034). The rest of the associations were not significant.

**Table 6:** The Impact of Coffee and Other Determinants on Sleep Quality* * The remaining variables were tested but were statistically non-significant, so they were not included. Significant values are indicated in bold. CI: confidence interval; SQS: Sleep Quality Scale; DM: diabetes mellitus; HTN: hypertension; BMI: body mass index

	SQS score				
Predictors	Beta		95% CI	p	
Coffee consumption (reference: non-consumer)					
Coffee (Yes, daily)	1.66		-2.34 to 5.67	0.415	
Coffee (Yes, regularly but not daily)	2.72		-0.99 to 6.43	0.151	
Coffee drinking time (No specific time)	-1.84		-4.76 to 1.08	0.216	
Coffee drinking time in the evening	-1.27		-4.59 to 2.06	0.454	
Coffee drinking time in the morning	-1.06		-4.28 to 2.15	0.516	
Gender (reference: female)					
Male	-4.17		-6.46 to -1.88	<0.001	
Age	-0.09		-0.22 to 0.04	0.187	
Occupation (reference: unemployed)					
Military	6.95		1.14 to 12.76	0.019	
Health sector	1.66		-2.44 to 5.76	0.426	
Agricultural sector	-12.56		-26.26 to 1.14	0.072	
Educational sector	1.85		-1.66 to 5.35	0.301	
Handicrafts and freelancing	-1.36		-13.63 to 10.91	0.827	
Industrial sector	-3.41		-14.05 to 7.24	0.530	
Administrative sector	-3.58		-9.50 to 2.33	0.235	
Another sector	1.91		-3.93 to 7.75	0.521	
Student	-2.32		-6.00 to 1.36	0.216	
Marital status (reference: single)					
Married	-2.51		-5.95 to 0.94	0.153	
Divorced/widow	-6.10		-12.62 to 0.41	0.066	
Device usage	0.39		0.15 to 0.62	0.001	
BMI	-0.05		-0.19 to 0.10	0.534	
Smoking history (reference: non-smoker)					
Current	-4.19		-9.13 to 0.75	0.096	
Ex-smoker	4.58		1.12 to 8.05	0.010	
Income					
Income (5000-9999 riyals)	-0.32		-3.51 to 2.87	0.844	
Income (10000-14999 riyals)	1.44		-1.54 to 4.41	0.343	
Income (More than 15000)	-0.32		-3.51 to 2.87	0.844	
Physical activity (reference: no physical activity)					
Engaging in moderate or vigorous physical activity for at least 30 minutes, 5 times a week.	-0.99		-3.22 to 1.25	0.387	
Engaging in moderate or vigorous physical activity for less than 30 minutes, 5 times a week.	-2.22		-4.84 to 0.39	0.095	
Diet (reference: no specific diet)					
Healthy diet	0.65		-1.70 to 2.99	0.588	
Chronic disease (reference: no chronic disease)					
DM (Yes)	-0.64		-4.38 to 3.10	0.737	
HTN (Yes)	1.09		-2.52 to 4.70	0.554	
Khat history (reference: non-user)					
Current user	4.84		0.37 to 9.30	0.034	
Former user	3.01		-1.49 to 7.50	0.189	
Other caffeinated drinks (references: non-consumer)					
Soft drinks (Always)	1.84	-1.22 to 4.91			0.238
Soft drinks (Sometimes)	-0.76	-3.28 to 1.77			0.555
Energy drinks (Always)	2.06	-2.14 to 6.27			0.336
Energy drinks (Sometimes)	1.25	-1.04 to 3.55			0.285
Tea (Always)	3.09	-0.70 to 6.88			0.109
Tea (Sometimes)	1.39	-2.32 to 5.10			0.463
Observations	824				

## Discussion

In this cross-sectional analysis of 824 participants, coffee consumption was highly prevalent (84%) but showed no significant association with SQS scores. Daily intake (β = 1.66, 95% CI: -2.34 to 5.67, p = 0.415), regular non-daily intake (β = 2.72, 95% CI: -0.99 to 6.43, p = 0.151), and timing of consumption all yielded non-significant results, suggesting no evidence of association in this sample while acknowledging the limits of measurement error, residual confounding, and reverse causation. In contrast, several variables were significantly related to poorer sleep quality, including female sex (β = -4.17, 95% CI: -6.46 to -1.88, p < 0.001), military occupation (β = 6.95, 95% CI: 1.14 to 12.76, p = 0.019), longer daily device use (β = 0.39, 95% CI: 0.15 to 0.62, p = 0.001), ex-smoking (β = 4.58, 95% CI: 1.12 to 8.05, p = 0.010), and current khat use (β = 4.84, 95% CI: 0.37 to 9.30, p = 0.034). Although modest in size, the device-use effect may be clinically relevant, given the mean of 7.7 hours/day. These findings suggest that occupational, behavioral, and stimulant-related factors may play a more dominant role in shaping sleep quality than coffee intake.

Coffee intake is considerably higher than global trends. In the USA, Wang et al. reported a coffee consumption rate of 52% while Rehm et al. found that the prevalence is 59% [17,18]. Similarly, in Bahrain, Jahrami et al. reported 76% coffee consumption among university students, a percentage still lower than our findings [19]. Regarding studies conducted in Saudi Arabia, Alfawaz et al. conducted a study in 2024 and reported an 88.2% coffee intake prevalence among female students, which aligns with our findings [20]. In addition, AlMutairi et al. found that 90% of participants consumed caffeinated drinks, with 74.63% being coffee drinkers [4]. This proportion was slightly lower than our findings, likely due to differences in sample size and sex distribution.

Our study did not find a noteworthy relationship between coffee intake and the quality of sleep. This is consistent with Del Brutto et al., who studied 716 participants from a rural Ecuadorian village and found that 72% had good sleep quality despite caffeine consumption (the average exposure effect: 0.027, 95% CI: -0.284 to 0.338, p = 0.866) [21]. Furthermore, Watson et al. reported similar findings in Australian adults, with 85% having good sleep quality regardless of coffee intake [22]. Conversely, in 2024, Khan and Alqurashi reported a considerably negative association between coffee consumption and sleep quality in research carried out in Makkah, Saudi Arabia. Using the PSQI, they found a median caffeine consumption of 324.20 mg per day, with 43.1% of participants exceeding the 400 mg recommended daily intake. Their study highlighted that higher caffeine intake correlated with poorer sleep quality [10,11]. This discrepancy might be due to demographic differences, caffeine intake level variations, or assessment tool differences.

Our finding that males have enhanced sleep quality compared to females is reinforced by earlier research indicating that lifestyle, stress exposure, and hormonal factors contribute to gender-based sleep disparities, conducted by Krishnan and Collop in 2006 [23]. The correlation between military occupations and poor sleep quality is consistent with a study carried out in 2020 by Chaves and Shimizu, suggesting that rigorous schedules and physical demands affect sleep efficiency [24]. Similarly, our findings that prolonged electronic device usage negatively impacts sleep quality align with research published in 2011 by Cajochen et al., who observed that exposure to blue light inhibits the release of melatonin [25].

Our study also found that former smokers reported poorer sleep quality compared to current smokers and non-smokers, a pattern that may be due to withdrawal symptoms or residual effects of past smoking. Similarly, Jaehne et al. reported that nicotine impacts sleep quality [26]. Lastly, our finding that khat use is associated with poor sleep quality is supported by Al-Motarreb et al., who identified cathinone as a stimulant capable of disrupting sleep patterns [27].

The high prevalence of coffee consumption in our sample can be attributed to cultural and environmental factors. Saudi coffee is a traditional beverage commonly served with desserts, and the rapid expansion of coffee shops in recent years has increased accessibility and affordability. Additionally, coffee consumption patterns vary based on situational factors such as academic exams, during which students tend to consume more caffeine. The lack of a noteworthy correlation between coffee intake and sleep quality suggests that other lifestyle factors may have a stronger influence on sleep. Stress levels, work schedules, and cultural sleep habits likely play a more dominant role in determining sleep quality than coffee consumption alone [5]. The gender-based differences in the quality of sleep observed in our research may be linked to hormonal fluctuations in women and increased caregiving responsibilities. Similarly, the poor sleep quality reported among military personnel is likely due to high occupational stress and demanding schedules. Our findings regarding the negative impact of electronic device use on the quality of sleep can be elucidated by the role of blue light in suppressing melatonin production, a mechanism well-documented in the literature. The association between khat use and poor sleep quality aligns with the stimulant effects of cathinone, which can delay sleep onset and lessen overall sleep duration.

Our findings suggest that coffee consumption is highly prevalent but does not significantly impact sleep quality. However, several other factors, such as gender, occupation, khat use, and electronic device usage, were found to influence sleep quality. For military personnel, targeted interventions such as educational programs on sleep hygiene, structured time management strategies, and medical consultations for individuals experiencing insomnia should be implemented. Similarly, awareness campaigns highlighting the adverse impact of extended electronic device use on sleep quality, through public health initiatives and medical counseling, could help mitigate its impact. The observed gender differences in sleep quality suggest the need for tailored approaches in addressing sleep-related issues in men and women. Women's sleep disturbances may be linked to caregiving responsibilities and hormonal fluctuations, necessitating gender-specific strategies for sleep improvement. Additionally, considering the correlation between khat use and poor sleep quality, public health initiatives should educate individuals about the possible negative effects of khat intake on sleep.

This study had several strengths, including its large sample size (n = 824), the high prevalence of coffee consumption in a culturally specific population, and the use of a validated sleep quality instrument (SQS) applied as a continuous outcome to enable regression modeling. The inclusion of relevant sociodemographic and behavioral variables, such as smoking, shisha, vaping, nicotine pouches, and lifestyle factors, provided region-specific insights. Nonetheless, limitations should be acknowledged. The cross-sectional design prevents causal inference, and measurement error in self-reported coffee intake may have occurred despite using a standardized cup size. The high proportion of “good sleepers” (92%) suggests possible misclassification or lenient cut-offs, although distribution plots were provided to aid interpretation. Selection bias is also possible due to electronic recruitment, which may underrepresent older or less internet-active individuals, and residual confounding by unmeasured factors (e.g., sleep disorders, medications, chronotype) cannot be excluded.

## Conclusions

This study found a high prevalence of coffee intake among adults in the Jazan region but no evidence of a significant association between coffee consumption and sleep quality in this cross-sectional analysis. However, factors such as gender, military occupation, electronic device use, smoking history, and khat consumption were significantly related to sleep quality. These findings suggest that lifestyle, behavioral, and occupational factors may exert a stronger influence on sleep outcomes than coffee intake alone. Given the study’s cross-sectional design, causal inference is not possible, and reverse causation remains plausible. Future research should incorporate objective measures of caffeine exposure and sleep quality, examine potential dose-response relationships, and explore emerging behaviors such as vape use and their impact on sleep, particularly among young adults.
